# Lipopolysaccharide-Induced M2 to M1 Macrophage Transformation for IL-12p70 Production Is Blocked by *Candida albicans* Mediated Up-Regulation of EBI3 Expression

**DOI:** 10.1371/journal.pone.0063967

**Published:** 2013-05-27

**Authors:** Xing-Feng Zheng, Yu-Xiao Hong, Gui-Jie Feng, Gao-Feng Zhang, Helen Rogers, Michael A. O. Lewis, David W. Williams, Zhao-Fan Xia, Bing Song, Xiao-Qing Wei

**Affiliations:** 1 Tissue Engineering and Reparative Dentistry, School of Dentistry, Cardiff University, Heath Park, Cardiff, United Kingdom; 2 Burn Unit, Changhai Hospital, Second Military Medical University, Shanghai, China; 3 Department of Dermatology, No. 1 Hospital of China Medical University, Shenyang, China; 4 School of Bioscience, Cardiff University, Cardiff, United Kingdom; UTHealth Medical School, United States of America

## Abstract

Macrophages are heterogeneous cell populations that are present in all tissues. Macrophages can be divided into classically activated inflammatory macrophages (M1) and alternatively activated anti-inflammatory macrophages (M2). It has been generally accepted that M1 macrophages are polarised in an inflammatory environment to produce pro-inflammatory cytokines, whilst M2 macrophages are involved in anti-inflammation and aid tissue repair in wound healing. Bacterial endotoxin (lipopolysaccharide; LPS) is a potent factor in infection, which induces M1 macrophages resulting in higher levels of iNOS, TNFα and IL-12p70 which dictate inflammatory T cell responses. M2 macrophages can be transformed into M1 macrophages following LPS stimulation to promote inflammation. *Candida albicans* is a commensal fungal microorganism, which has been suggested to induce immune tolerance; however, the mechanism of *C. albicans-*induced immune tolerance has not been investigated in detail. IL-35 is a recently identified anti-inflammatory cytokine which is a heterodimeric protein consisting of the Epstein-Barr virus-induced gene 3 (EBI3) and IL-12p35. IL-35 shares the protein subunit p35, with IL-12p70. IL-12p70 is the most potent cytokine to induce Th1 responses during inflammation. In this study, we demonstrate that heat-killed *C. albicans* (HKC) strongly suppressed LPS-induced IL-12p70 production in M2 macrophages. *Candida albicans* induced a high level of EBI3 expression in M2 macrophages, which served as a mechanism for IL-12p70 suppression by competitive binding of the common protein subunit (p35) of IL-35 and IL-12p70. To demonstrate that EBI3 expression had the ability to block IL-12p70 production intracellularly, a Chinese Hamster Ovary (CHO) cell line with biscistronic expression of IL-12p40 and p35 was constructed, followed by ectopic over-expression of EBI3. The over-expression of EBI3 in the IL-12p70 producing cell line effectively suppressed IL-12p70 production. IL-35 secretion was also detected in the cell line, with suppressed IL-12p70 production by immune-precipitation Western blotting. However, this secretion was not evident in M2 macrophages following stimulation by HKC. This can be explained by the constitutive expression of IL-35 receptors (gp130 and IL-12Rβ2) in M2 macrophages for cytokine consumption. Our results have indicated that *C. albicans* can suppress host inflammatory responses in mucosal skin by suppressing LPS-induced IL-12p70 production. Lower IL-12p70 production may avoid an unnecessary Th1 response in order to retain immune tolerance, which may be one of the mechanisms by which *C. albicans* achieves a successful commensal lifestyle without having a detrimental effect on the host’s health.

## Introduction

Macrophages are dispersed throughout the body and represent a highly heterogeneous cell population which demonstrate plasticity in their cell polarisation. Macrophages play an important role in immune surveillance [1], [2], as they produce cytokines to alert host immune cells and can also be phenotypically transformed by a tissue environment. It has been widely accepted that at least two types of macrophages, with distinct phenotypes, can be found in the processes of infection and wound healing: classically activated macrophages (M1) and alternatively activated macrophages (M2) [3], [4], [5]. In an inflammatory environment, inflammatory T cells, such as Th1 and Th17, produce Granulocyte-Macrophage Colony-Stimulating Factor (GM-CSF) which drives macrophage maturation. GM-CSF together with bacterial lipopolysaccharide (LPS) induce inflammatory macrophages (M1) from bone marrow-derived macrophages (BMDM) which exhibit high levels of iNOS, IL-12 and TNFα production [6]. In contrast, M2 macrophages can be generated *in vitro* by culturing BMDM with macrophage colony-stimulating factor (M-CSF) together with IL-4. M2 cells predominantly produce higher levels of arginase-1 and IL-10 [6], [7]. Macrophages switch their phenotypes depending on the tissue environment [8], [9], [10] and this phenotypic plasticity is of benefit to both the host and pathogen, since inappropriate host responses can result in tissue damage which may lead to chronic wounds and pathogen invasion.

*Candida albicans* is an opportunistic fungal pathogen, capable of causing life-threatening infections in immune incompetent individuals [11], [12]. However, *C. albicans* is found on moist mucosal surfaces in the majority of healthy humans, without causing disease and this commensal existence has been associated with host immune tolerance. *Candida albicans* has two basic morphological growth forms, namely hyphae and yeast. It is often stated that hyphae on mucosal surfaces induce an immune response, whilst a predominant yeast presence is linked to a commensal existence on the mucosal surface and may be more potent at inducing immune tolerance. A fine balance between host immune responses to *C. albicans* and bacterial LPS stimulation may play a key role in maintaining healthy mucosal surfaces; however, the detailed mechanism of *C. albicans* inducing immune tolerance has not been extensively studied.

Macrophages are a major cell source for production of the IL-12 family of cytokines during infection and inflammation. IL-12 consists of two protein subunits, IL-12p40 and p35, forming a heterodimeric cytokine IL-12p70, which is an effective cytokine in driving Th1 responses. IL-35 shares the IL-12p35 subunit and is heterodimeric with EBI3. IL-35 is one of the anti-inflammatory cytokines that promotes regulatory T cells (Treg) to suppress immune response [13], [14], [15]. The macrophage phenotypes are largely dependent on cytokine production in the tissue environment. Switching a macrophage from an inflammatory cytokine producing cell to an anti-inflammatory producing cell may hold the key to induced non-immune responses or peripheral tolerance.

In this study, we found that LPS induced an M2 to M1 cell phenotype transformation, thereby inducing IL-12p70 production. Heat-killed *C. albicans* (HKC) suppressed LPS-induced IL-12p70 production in a dose-dependent manner in M2 macrophages and this inhibition was associated with induced EBI3 expression, a subunit of IL-35. Over-expression of EBI3 in an IL-12p70 expression cell line reduced IL-12p70 production and detectable secretion of IL-35. This result demonstrated that *C. albicans* induced anti-inflammatory cytokine IL-35 production in M2 macrophages and blocked LPS-induced M2 to M1 macrophage phenotype transformation. We postulate that this serves as the mechanism behind *C. albicans-*induced immune tolerance.

## Materials and Methods

### M1 and M2 Macrophages Culture and Stimulation

The protocols for animal handling were previously approved by our institutional Animal Ethics Committee according to UK Home Office guidance. Bone marrow cells were harvested from the femurs of male C57/Black mice and prepared accordingly for culture of bone marrow M1 (classical) and M2 (alternative) macrophages using 10 ng/ml recombinant GM-CSF (ImmunoTools) and M-CSF respectively (ImmunoTools) [9]. Briefly, bone marrow cells were cultured in RPMI 1640 containing 10% (w/v) foetal bovine serum (FBS), 100 U/ml penicillin/streptomycin (Invitrogen, Glasgow, UK) and either GM-CSF or M-CSF. The medium was changed every other day for 7 days. Harvested M1 and M2 macrophages were seeded at a density of 1×10^5^ cells/well in a 24 well plate, then cultured with full medium for 24 h. To fully polarise M1 and M2 macrophages, GM-CSF derived M1 macrophages were stimulated with 10 ng/ml bacterial LPS and M2 macrophages were cultured in M-CSF with 10 ng/ml IL-4 (ImmunoTools). In certain experiments, macrophages were stimulated with increasing concentrations of heat-killed *C. albicans* (HKC). The cell lysate was harvested at the indicated time points for RNA extraction for quantitative RT-qPCR. Cell lysates and culture supernatants were also harvested at the indicated time points for Western blotting and an enzyme-linked immunosorbent assay (ELISA). To block EBI3 protein transportation, 3 µg/ml of Brefiedin A (eBiosciences Ltd, USA) was added to the cell cultures 1 h before cell harvesting.

### Preparation of Heat-killed *C. albicans*

A well characterised clinical isolate of *C. albicans* was used for the challenge studies [16]. This isolate had previously been identified based on traditional biochemical analysis as well as sequencing of rDNA gene sequences. The isolate was cultured overnight at 37°C in Yeast Nitrogen Base medium supplemented with 0.5% v/v glucose. The cells were harvested by centrifugation at 3000×*g* and washed three times with phosphate buffered saline (PBS) before being heated at 98°C for 10 min. Yeast viability was then assessed by culture on Sabouraud dextrose agar to confirm total cell death.

### RT-qPCR to Detect mRNA Expression in Mouse Macrophages

To detect the expression of mouse IL-12p40, p35 and EBI3 as well as iNOS and Arginase 1, total RNA was extracted from lysed cells using RNeasy (QIAGEN) following the manufacturer’s instructions. The RNA yield and purity were measured using a spectrometer (nanodrop), and 1 µg of each RNA sample was used for reverse transcription of cDNA. The mRNA levels of mouse iNOS, Arginase-1, IL-12p35 and EBI3 as well as IL-12Rβ2 and gp130 (IL-35 receptor chains) [17] and TLR4 were compared with the housekeeping genes β-actin or mHPRT. The following primer pairs were used: mouse iNOS sense, 5′- TGG CTC GCT TTG CCA CGG ACG AGA CGG A and anti-sense, 5′- GGA GCT GCG ACA GCA GGA AGG CAG CGG G. Mouse Arginase-1 sense, 5′- AGCT GGC TGG TGT GGT GGC AGA GGT CCA, and anti-sense, 5′- GGG TGG ACC CTG GCG TGG CCA GAG ATG CT. Mouse IL-12p35 sense, 5′- CCC TTG CAT CTG GCG TCT ACA CTG CTG C, and anti-sense, 5′- AGG AGG GCA AGG GTG GCC AAA AAG AGG A. Mouse EBI3 sense, 5′- GCC TCC TAG CCT TTG TGG CTG AGC GAA T, and anti-sense, 5′- AGA GAG AAG ATG TCC GGG AAG GGC CAG. Mouse IL-12Rβ2 sense, 5′- CAG GGA GCA TCA CGA AGT TTC CCC CAC A and anti-sense, 5′- TTT GTG CTT GGA GTC ACC CCG GAT GGA G. Mouse gp130 sense, 5′- CAG GAA GAC GCT ACC GTG AAT CGG ACC C, and anti-sense, 5′- CTC TGG CAG GAG CGG CTT GTT TGA GGT A. Mouse TLR4 sense, 5′- GAC TCT GAT CAT GGC ACT GTT CTT CTC C and anti-sense; 5′- CAG GGA CTT TGC TGA GTT TCT GAT CCA T. Mouse β-Actin sense, 5′- TCT TTG CAG CTC CTT CGT TGC CGG TCC, and anti-sense, 5′-GTC CTT CTG ACC CAT TCC CAC CAT CAC AC. Mouse HPRT sense, 5′-TTG ATT GTT GAA GAT ATA ATT GAC ACT and anti-sense, 5′-TTC CAG TTT CAC TAA TGA CAC A.

PCR products were obtained using the following thermal cycles, 95°C for 15 seconds, 60°C for 30 seconds and 72°C for 30°C for 40 cycles. RT-qPCR results were visualised using a 1.5% (w/v) agarose gel containing ethidium bromide. To quantify the mRNA levels present in M1 and M2 macrophages, relative gene expression levels were determined using a SYBR Green qPCR kit (Sigma). All samples were run in triplicate. The CT value of each sample was acquired following a calculation of the 2^−ΔΔ^Ct, and all data were expressed as a percentage of gene/β-actin (%).

### ELISA for Mouse IL-12p70 and TNFα

IL-12p70 and TNFα commercial ELISA kits (eBiosciences) were used to detect IL-12p70 heterodimer protein and TNFα in cell culture supernatants. Briefly, the wells of a high protein binding 96-well plate (Greiner Bio-one Ltd) were coated overnight with 50 µl monoclonal anti-mouse cytokine antibody (IL-12p70 or TNFα). After blocking non-specific binding with 10% (w/v) FBS in PBS for 2 h at 37°C, clear cell supernatants (50 µl) and a concentration series of recombinant mouse IL-12p70 or TNFα were added to the plate in triplicate before incubating at 37°C for 2 h. Between incubation steps, wells were washed thoroughly with ELISA wash buffer (PBS containing 0.05% (v/v) Tween-20 [Sigma]). Bound cytokines were detected using a biotin-conjugated antibody followed by StreptAvidin-Horse radish peroxidase (HRP), with 1 h incubation at 37°C. HRP was finally revealed with 3,3′,5,5′-Tetramethylbenzidine (TMB) and the reaction stopped by adding 50 µl TMB-Stop solution. The concentration of cytokines was determined using an ELISA plate reader.

### Western Blotting and Immune Precipitation Western Blotting

Intracellular protein expression was determined by Western blotting under reducing conditions. Briefly, the protein concentration of the cell lysates was measured using the bicinchoninic acid assay (BCA; Pierce), using bovine serum albumin (BSA) as a standard. Cell lysates containing 20 µg of total protein were resolved by SDS-PAGE on a 4–20% tris-glycine gradient gel (NuPAGE, Novex, Invetrogen) before being transferred to a nitrocellulose membrane in transfer buffer (Invitrogen) at room temperature (RT). The membrane was blocked with 10% (w/v) milk powder at RT for at least 1 h before probing the protein bands with specific antibodies against EBI3 (eBioscience) and/or p35 (R&D system). The expression level of the β-actin housekeeping gene was used for equal loading control in all experiments.

Immune precipitation Western blotting was performed using the laboratory’s standard protocol. Briefly, 0.5 ml of clear cell lysate or 1 ml of cell culture supernatant was pre-treated with 20 µl protein-A agarose beads for 30 min at room temperature. After this, 2–5 µg of anti-IL-12p35 antibody (R&D system) was added to each tube and incubated for at least 1 h at room temperature on a rotating mixer. After IL-35 (EBI3/p35) and anti-p35 antibody binding, 20 µl of protein A agarose beads were added to each tube and incubated for 1 h at room temperature, or overnight at 4°C. The beads were then washed three times with PBS containing 0.05% (v/v) Tween-20. The precipitated protein was eluted by adding 50 µl of protein loading buffer and heating at 95°C for 5 min. The eluted protein was loaded on to an SDS-PAGE gel for Western blotting with an anti-EBI3 antibody (eBioscience) as described earlier.

### Mouse EBI3 mRNA Knockdown by esiRNA in RAW264.7 Cells

Endoribonuclease-prepared short interfering RNAs (esiRNA) specific for mouse EBI3 gene were obtained from Sigma (Cat No; EMU059781-20UG). The mouse macrophage cell line (RAW264.7) was cultured with 20 ng/ml each of M-CSF and IL-4 for 4 days for M2 maturation prior to cells being transfected with increasing concentrations of mouse EBI3 specific esiRNA and a sham transfection as a negative control (-ve) with Lipofectamine2000 (Invitrogen). The cells were stimulated with 10^6^ HKC after 24 h cell transfection. Cells were harvested for RNA extraction after 6 h HKC stimulation. Quantitative RT-PCR was performed as described above to examine EBI3 mRNA levels. In another experiment, cells were transfected with or without the esiRNA followed by stimulation with HKC for 48 h. The cell culture supernatants were collected to examine the levels of IL-12p70 production by ELISA (eBioscience).

### Mouse IL-12p40/p35 Biscistronic Expression and EBI3 Vector for Protein Expression

Mouse IL-12p40, p35 and EBI3 cDNAs were cloned via RT-PCR with cDNA extracted from LPS stimulated J774 cells as previously described [14]. The cDNAs containing the open reading frames (ORFs) of IL-12p40 and p35 were inserted into a pcDNA3.1A-IRES vector to give the plasmid pC3-p40-IRES-p35. To establish Chinese Hamster Ovary (CHO) clones with IL-12p40/p35 biscistronic expression, the cells were transfected with 1 µg of pC3-p40-IRES-p35 plasmid DNA followed by selection of G418 resistant clones over a 14-day culture period. IL-12p40/p35 expressing CHO clones were identified and expanded in culture. Functional IL-12p70 production by the CHO cell clones was confirmed by stimulation of IFNγ production in purified CD4^+^ T cells from a mouse spleen. The EBI3 expression vector was constructed through insertion of EBI3 cDNA into pcDNA4/TO-A. EBI3 expression in CHO cells was also demonstrated by Western blotting with antibodies against EBI3.

### Detection of Memory Bound EBI3 on Macrophage Cell Surface by FASC Analysis

The Mouse RAW264.7 macrophage cell line was cultured in the M2 macrophage conditional medium (RPMI 1640 full medium containing 20 ng/ml of M-CSF and 20 ng/ml of IL-4) for 4 days. After this time, 5×10^5^ cells per well of a 6-well plate were incubated overnight with or without 10^6^ HKC/ml. The macrophages were then harvested and washed with cold PBS before staining with anti-mouse EBI3 antibody (eBioscience) or isotype control antibody, followed by PE-Texas red-labelled secondary antibody. Cell surface bound EBI3 was then detected by FACS analysis.

### Statistical Analysis

Results were calculated as means ± standard deviations (SD). Statistical significance was determined using a one-way ANOVA and Tukey-Kramer or Bonferroni multiple comparisons post-tests to analyse differences between groups; P<0.05 was considered significant.

## Results

### Phenotypes of M1 and M2 Macrophages Derived from Mouse BMDM

**Figure 1:**
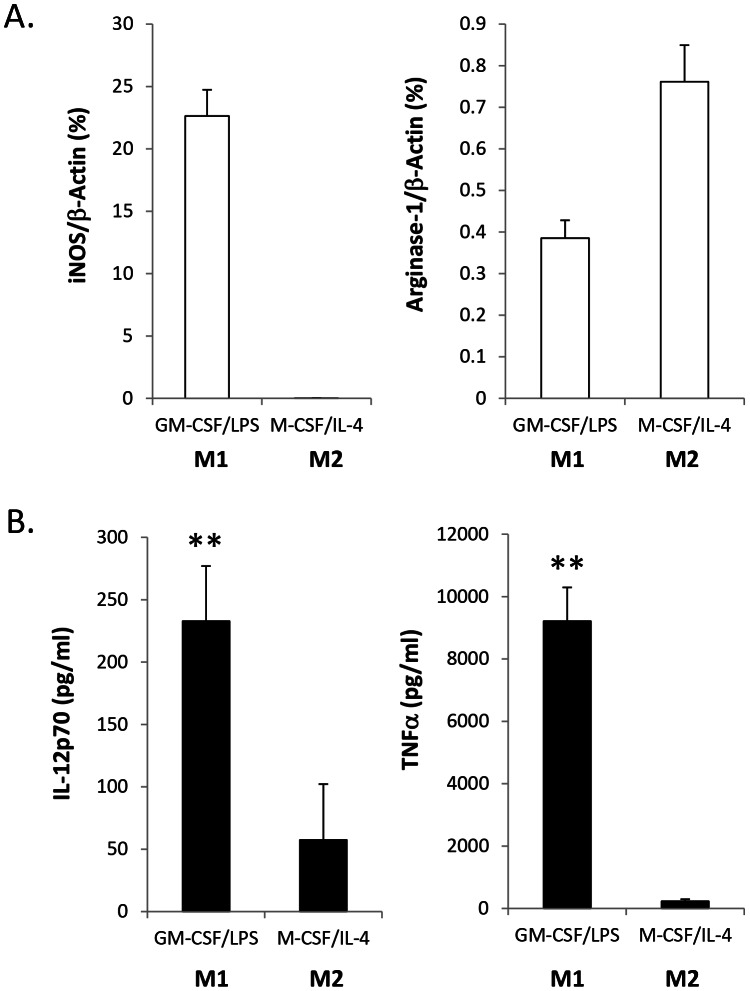
M1 and M2 gene expression in macrophages. Mouse bone marrow cells from C57/black mice were differentiated using 10 ng/ml GM-CSF and 10 ng/ml (each) of M-CSF/IL-4 for M1 and M2 macrophages, respectively for 7 days prior to overnight stimulation with or without LPS (10 ng/ml). **A.** Lysed cells were used for quantitative real time PCR (RT-qPCR) to quantify gene expression of iNOS and arginase-1 compared with β-actin gene expression. **B.** The production of TNFα and IL-12p70 was examined by ELISA. The results are representative of three independent experiments.

To demonstrate the phenotypes of M1 and M2 macrophages generated in our culture conditions, iNOS Arginase-1 mRNA expression in M1 and M2 macrophages were examined by RT-qPCR. M1 macrophages cultured in GM-CSF/LPS expressed much higher levels of iNOS mRNA compared with M-CSF/IL-4 cultured M2 macrophages, whilst a higher level of arginase-1 expression was detected with M2 macrophages when compared with M1 macrophages (Figure 1A). Significantly higher levels of IL-12p70 and TNFα were also detected with M1 macrophages, but not with M2 macrophages (Figure 1B). This result indicated that successful polarisation of M1 and M2 from BMDM was achieved in the culture conditions used.

### *Candida albicans* Suppresses LPS-induced IL-12p70 in M2 to M1 Phenotype Switching

**Figure 2:**
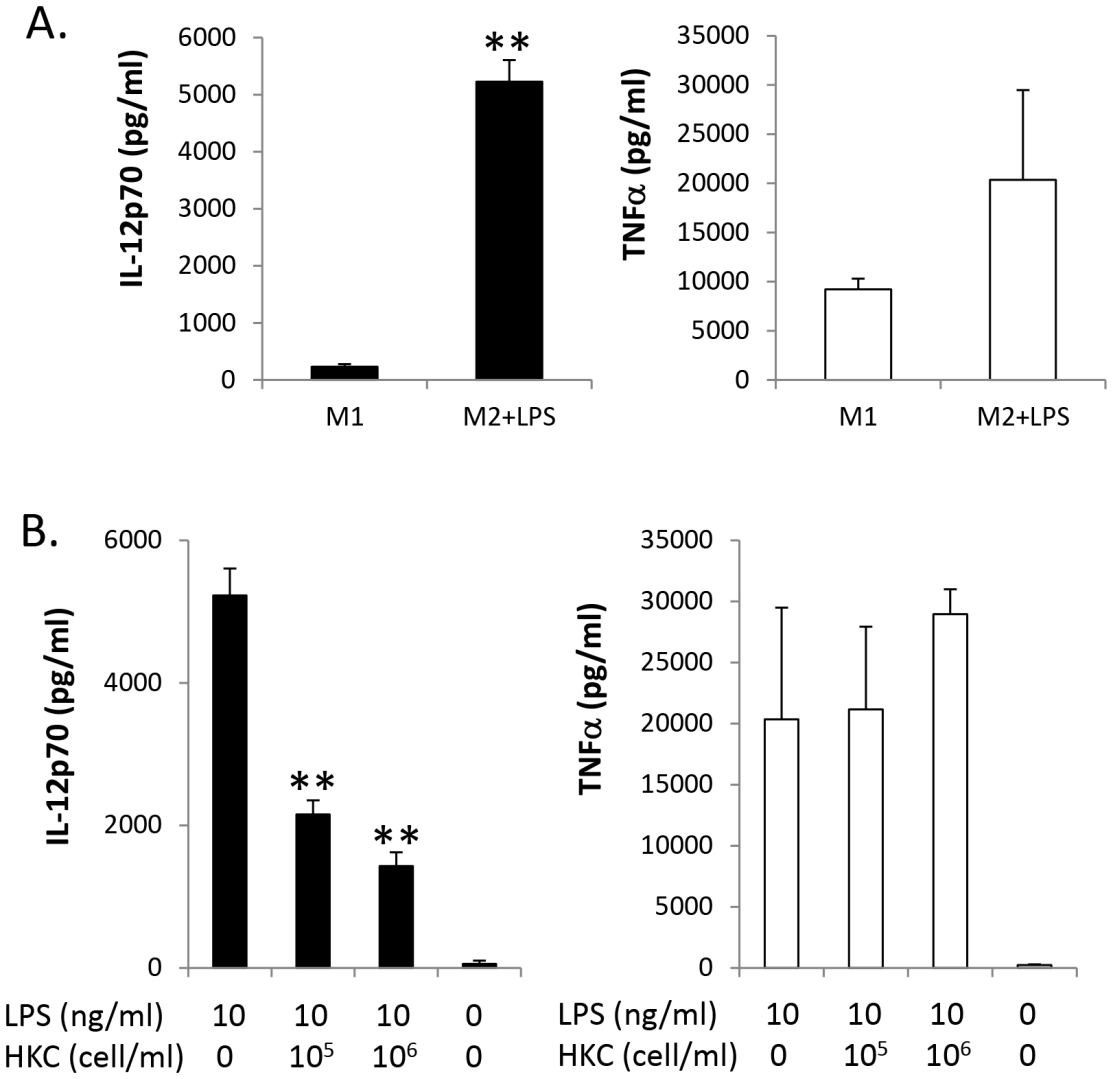
Heat-killed *C. albicans* inhibits LPS-induced M2 to M1 shifting for IL-12p70 production. **A.**α expression via ELISA. **B.** M2 macrophages were stimulated with increasing concentrations of HKC plus 10 ng/ml LPS. IL-12p70 and TNFα production was quantified by ELISA. The results are representative of three experiments. **p<0.01.

Macrophages demonstrate plasticity in phenotype switching. Inflammatory M1 macrophages can be polarised into M2 macrophages within tissues during the resolving stage of infection and wound healing. M2 macrophages produced lower levels of inflammatory cytokines, but higher anti-inflammatory cytokines and growth factors following HKC stimulation (data not shown). To demonstrate plasticity of macrophage polarisation, fully polarised M1 and M2 macrophage were stimulated with 10 ng/ml bacterial LPS and the cell culture supernatants harvested 48 h after stimulation. In these experiments, IL-12p70 and TNFα production was determined via ELISA. It was evident that following LPS stimulation, a 20-fold higher IL-12p70 expression in M2 occurred compared with M1 macrophages. However, TNFα production only increased 2-fold in M2 macrophages, with no significant difference when compared with M1 macrophages (GM-CSF/LPS) (Figure 2A). Using qRT-PCR a 3-fold greater TLR4 mRNA expression in M2 macrophages was evident compared with that in M1 macrophages (Figure S1). An increased concentration of HKC resulted in dose-dependent suppression of LPS-induced IL-12p70 production by M2 macrophages, but the levels of TNFα production did not alter in these cells (Figure 2B). Since IL-12p70 is a critical pro-inflammatory cytokine, which dictates Th1 polarisation, suppressing IL-12p70 production induced by LPS, may serve as a critical mechanism of by which *C. albicans* induces immune tolerance.

### *Candida albicans* Stimulates Increased Levels of EBI3 mRNA and Protein in M2 Macrophages

**Figure 3:**
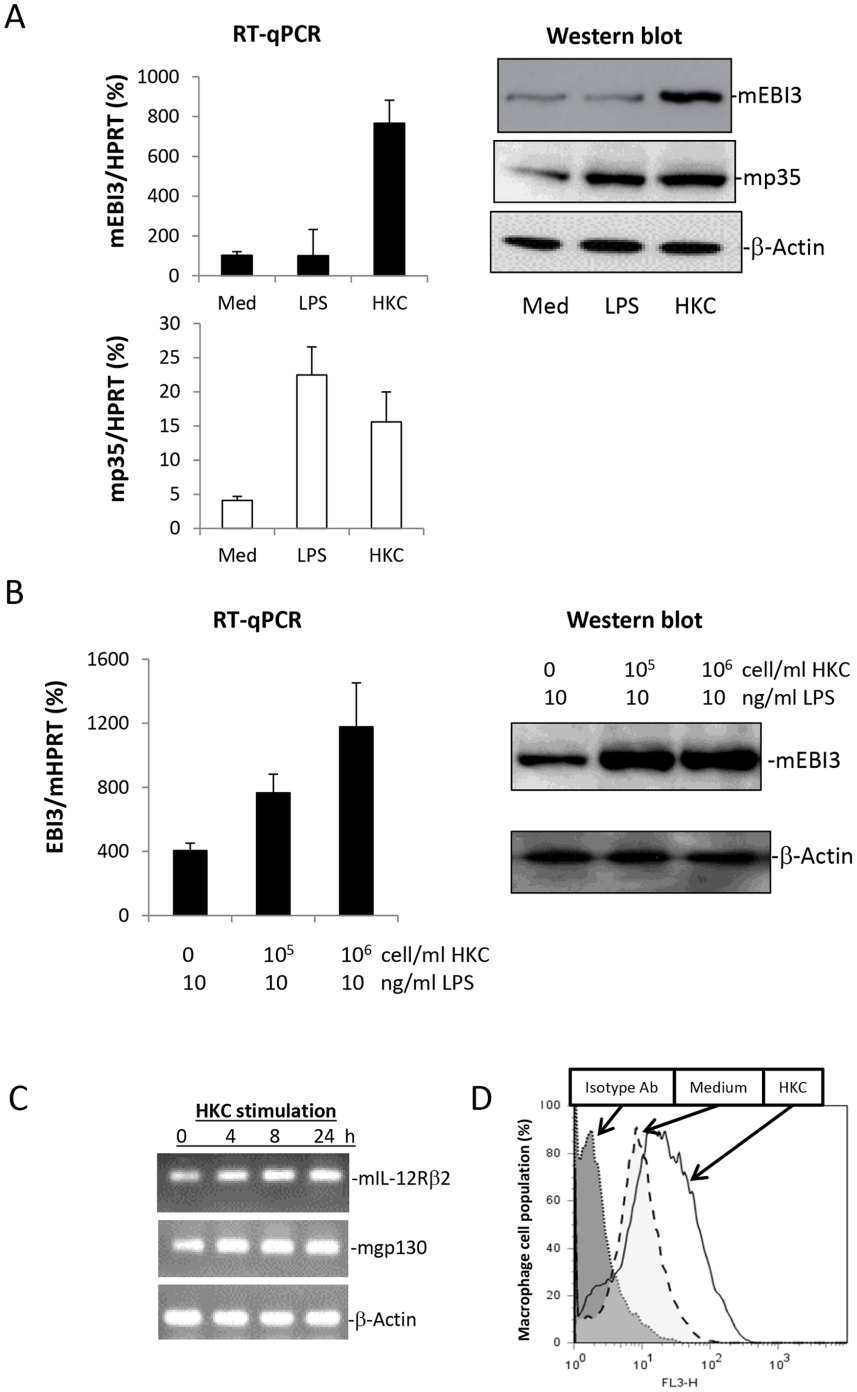
Heat-killed *C. albicans* enhances EBI3 expression in M2 macrophages with or without LPS stimulation and IL-35 receptors for cytokine consumption. **A.** M2 macrophages were stimulated with 10 ng/ml LPS or 10^6^ cells/ml HKC for 6 hours before lysing the cells for RNA extraction to detect relative levels of EBI3 and IL-12p35 mRNA expression relative to the HPRT mouse housekeeping gene, by RT-qPCR. The cells were stimulated overnight to detect EBI3 and p35 protein expression via Western blotting. **B.** M2 macrophages were stimulated with increasing amounts of HKC plus 10 ng/ml LPS for 6 hours. The relevant level of EBI3 expression was quantified by RT-qPCR against the HPRT housekeeping gene. EBI3 protein expression was visualised by Western blotting after overnight cell stimulation. The results are representative of two experiments. **C.** Mouse mIL-12Rβ2 and gp130 mRNA expression in M2 macrophages with or without HKC stimulation. The results are representative of 3 independent experiments. **D.** Detection of EBI3 on the cell surface of M2 macrophages with and without HKC stimulation.

Previously we reported that HKC induced a higher level of EBI3 expression in human monocytes/THP-1 cells [13]. In this study, we have confirmed that HKC stimulation results in much higher EBI3 levels in mouse BMDM compared with levels resulting from LPS stimulation. In contrast, LPS and HKC stimulation resulted in comparable levels of IL-12p35 expression (Figure 3A). In macrophages, EBI3 mRNA was expressed at much higher levels than p35 mRNA, which was demonstrated by the percentage expression of genes of interest compared with the mouse housekeeping gene, adenine phosphoribosyltransferase (HPRT) (Fig. 3A). Moreover, HKC was found to induce dose-dependent EBI3 expression at both the mRNA and protein level in M2 macrophages in the presence of LPS stimulation (Fig. 3B). Although we have detected expression of both IL-35 protein subunits in macrophages after stimulation by HKC, immune precipitation Western blotting failed to detect IL-35 secretion in cell culture medium (data not shown). However this can be explained by detecting IL-35 receptors expression in M2 macrophages (Figure 3C). The consumption of IL-35 by its receptors on M2 macrophages was further evidenced by detection of cell membrane bound EBI3 by FACS staining with anti-EBI3 antibody (Figure 3D). However expression of the IL-35 receptor did not trigger cell signalling to inhibit IL-12p70 production, since adding recombinant IL-35 or IL-27 to M2 macrophage cell cultures did not alter LPS induced IL-12p70 production (Figure S2). Furthermore, HKC induced EBI3 expression plays a role in suppression of LPS induced IL-12p70 production. This was supported by results obtained following EBI3 ‘knock down’ in the mouse macrophage cell line (RAW264.7) which rescued HKC inhibition of LPS-induced IL-12p70 production (Figure S3). Competition between EBI3 and IL-12p40 for IL-12p35 coupling was further confirmed by the following experiments.

### Over-expression of EBI3 in IL-12p70 Expressing CHO Cell Lines Suppresses IL-12p70 Production

**Figure 4:**
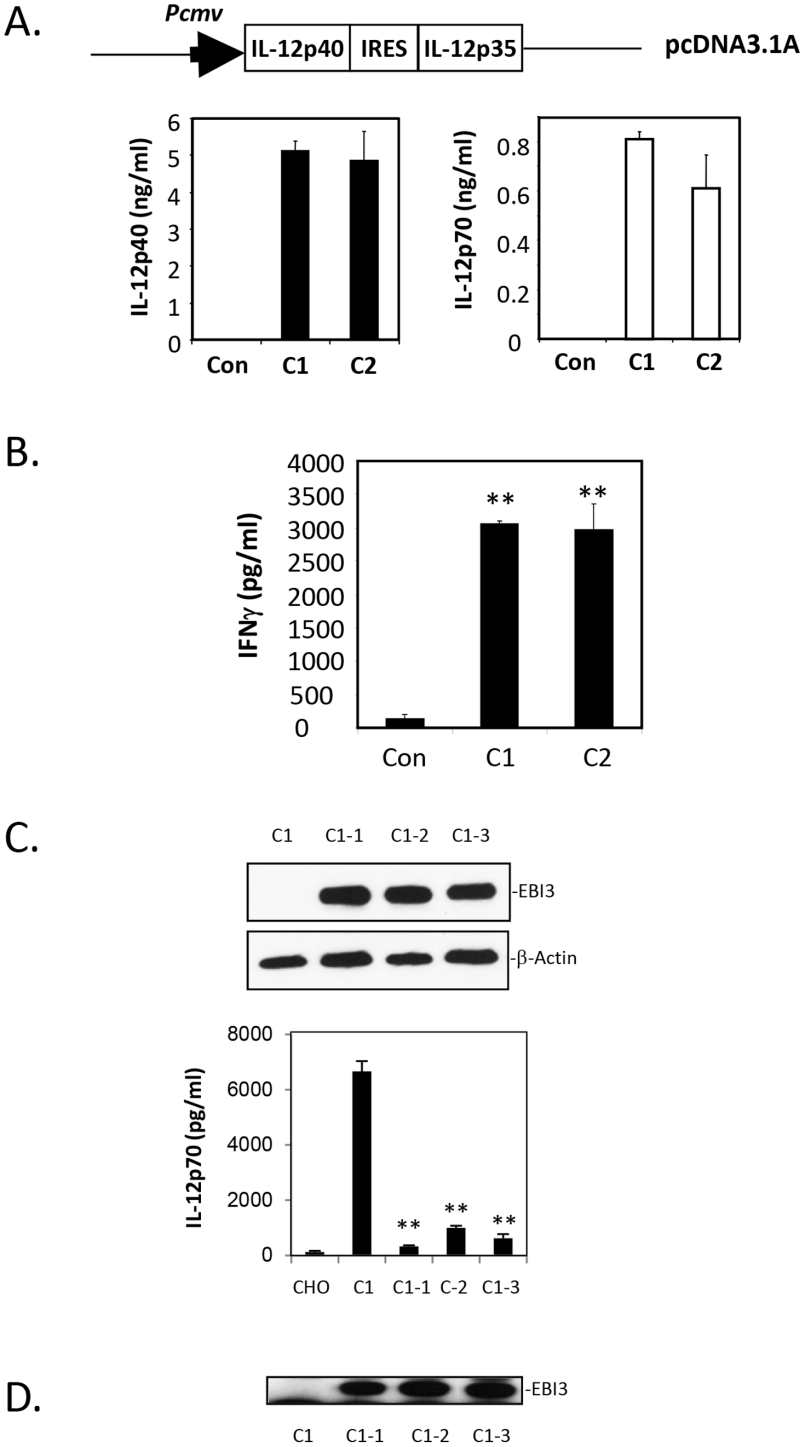
Over-expression of EBI3 blocks IL-12p70 production in CHO cells. **A.** Biscistronic IL-12p40 and p35 expression were compared before being introduced into a CHO cell by transfection. Two clones (C1 and C2) with comparable levels of IL-12p40 and IL-12p70 as detected by ELISA were selected. **B.** The biological activity of IL-12 was detected by inducing IFNγ production in C57/black mouse CD4^+^ T cells. **C.** The EBI3 expression plasmid was transfected into IL-12p70 expression clone 1 to detect EBI3 expression by Western blotting. Three clones (C1–1, C1–2 and C1–3) with high levels of EBI3 expression plus a non-transfected IL-12p70 expression clone (C1) were used to detect EBI3 expression by Western blotting. The culture supernatants from these 4 clones, plus wild type CHO cells were harvested for use in the IL-12p70 ELISA. **D.** The culture supernatants were also used to detect IL-35 (EBI3 and p35) heterodimeric protein secretion by immune precipitation Western blotting.

To further investigate the impact of EBI3 expression on IL-12p70 production, IL-12p70 producing CHO cell lines were established by transfection to give biscistronic expression of IL-12p40 and p35. Two clones with comparable levels of IL-12p40 and IL-12p70 expression were used for testing IL-12p70 biological activity using purified mouse CD4^+^ T cells (Figure 4A). Cell culture supernatants collected from both clones were able to stimulate IFNγ production (Fig. 4B). We selected one of the two clones for transfection with the EBI3 over expression vector. Three representative clones with higher EBI3 expression levels were identified following the detection of IL-12p40 and IL-12p70 in the culture medium. IL-12p70 producing CHO cells with over-expression of EBI3 showed a large reduction in IL-12p70 production (Fig. 4C) which was associated with detectable levels of IL-35 secretion in the culture supernatant (Fig. 4D). This result demonstrated that intracellular over-expression of EBI3 effectively suppressed IL-12p40 and p35 dimerisation for IL-12p70 production. This effect provides an intrinsic mechanism of regulation for the IL-12 family of cytokines, which serves to balance immune responses.

## Discussion

Host innate and adaptive immune cells orchestrate their development to control pathogen invasion and also limit tissue damage or promote tissue repair. *Candida albicans* is an opportunistic pathogen which is normally effectively controlled by the host immune system. One of damaging features of host immunity is its over-reaction in response to a pathogen. In a host-commensal *C. albicans* relationship, *C. albicans* is able to induce innate immune responses, but these are sufficiently mild so as not to trigger severe inflammation and tissue damage. The mechanism behind this fine balance of the host immune response has not been established in detail. Macrophages are one of the most important host innate immune cells and exist in all tissues including mucosal surfaces. Macrophages play a key role in sensing and controlling *C. albicans* and bacterial infections by phagocytosing the pathogen and releasing cytokines. Macrophages are heterogeneous in phenotype and exhibit plasticity in polarising to adapt to different tissue environments. Two types of macrophages have been identified in infection and tissue repair, namely M1 and M2 macrophages. M1 macrophages are usually associated with higher pro-inflammatory cytokine production, which initiates adaptive immune T cell responses and induces tissue inflammation by producing cytokines such as IL-12p70, IL-23 and TNFα [18], [19], [20], [21], [22], [23]. This inflammatory response promotes host defences against *C. albicans* and bacterial invasion of the mucosal epithelium. However, prolonged inflammatory cytokine production induced by M1 macrophages may lead to persistent inflammation as a result of excessive Th1 and Th17 responses. Although large numbers of Th1 and Th17 cells migrating into inflamed tissue help enforce macrophage killing, they also cause tissue damage [24], [25]. Resolving pathogen invasion and the initiation of tissue repair is associated with M2 macrophages through increased concentration of growth factors and release of anti-inflammatory cytokines into the tissues [26], [27]. M2 macrophages also exhibit phenotypic and functional plasticity [8].

Bacterial products, such as LPS, are potent bioactive factors which result in the switch of macrophage phenotype from M2 to M1 [9]. The aim of this study was to elucidate how *C. albicans* could potentially impact on these key innate immune cells (macrophages) in terms of response and tolerance through use of an *in vitro* macrophage culture model. We used GM-CSF to drive BMDM to become M1 macrophages. The development of classical M1 macrophages occurs in a tissue environment containing high levels of GM-CSF and IFN-γ produced by Th1 and Th17 cells [5], [9]. GM-CSF effectively induced IFN-β production in an autocrine manner, triggering STATA-1 signalling, which has the same effect as IFN-γ [28]. It is generally accepted that GM-CSF plus LPS produces M1 macrophage phenotypes *in vitro*. M2 macrophages were produced by culturing bone marrow macrophages in a medium containing M-CSF and IL-4. M1 and M2 macrophages generated in this study showed typical M1 and M2 phenotypes. M1 macrophages produced higher levels of iNOS, TNFα and IL-12p70, while M2 macrophages demonstrated higher levels of arginase-1 and vascular endothelial growth factor (VEGF), but lower levels of iNOS, TNFα and IL-12p70. Although M2 macrophages expressed a higher level of EBI3 than M1 macrophages, HKC stimulation further enhanced EBI3 expression in M2 macrophages.

Bacterial products (*e.g.* LPS) can induce inflammation and promote M1 macrophage polarisation. However, LPS can also cause M2 macrophages involved in wound healing to reverse to the inflammatory M1 phenotype, with a higher level of iNOS expression and pro-inflammatory cytokines, such as IL-12p70 and TNFα production [9], [29]. Although this M1 inflammatory macrophage effectively controls pathogen invasion, it can also impede the wound healing process and induce tissue damage [30], [31]. The appropriate transformation of macrophages from M1 to M2 phenotype in the process of resolving infection towards wound healing, is crucial for limiting tissue inflammation and promoting tissue repair. Maintaining the M2 macrophage phenotype is key for retaining immune tolerance. In general, *C. albicans* stimulated lower responses in macrophages compared with those following LPS stimulation. We found that HKC was not able to stimulate iNOS, TNFα or IL-12 production in M2 macrophages, but was able to with M1 macrophages. In contrast, LPS stimulated iNOS, TNFα and IL-12 production in both M1 and M2 macrophages to a different degree. However, HKC effectively suppressed LPS-induced IL-12p70 production only in M2 macrophages. *Candida albicans* stimulated high EBI3 expression by M2 macrophages which blocked switching of LPS-induced M2 to M1 cell phenotype with IL-12p70 production. Production of the IL-35 heterodimer protein (EBI3/p35) as detected by immune precipitation, was associated with decreased IL-12p70 secretion by transfected CHO cells. However, we failed to detect IL-35 production by M2 macrophages, which may be explained by induced expression of IL-12Rβ2 and gp130, a receptor for IL-35 (p35/EBI3). IL-35 receptor expression in M2 macrophage may bind to IL-35 and thereby restrict its secretion; this is supported by the detection of both EBI3 and p35 expression in lysed M2 macrophages and detection of increased cell surface bound EBI3 by FACS staining (Figure 3D).

To demonstrate an autocrine role of IL-35 on macrophages for LPS induced IL-12p70 production, we added increasing concentrations of either recombinant mouse IL-35 or IL-27 [14], [32] into M2 macrophages cell cultures, followed by LPS stimulation. This did not significantly alter the levels of IL-12p70 in M2 macrophages culture supernatant (Figure S2). This may indicate that IL-35 and IL-27 triggered cell signals, may not be involved in IL-12p70 gene transcription. Further study will examine the cell signalling activation by IL-35 or IL-27 in M2 macrophage and dendritic cells.

IL-27 also belongs to the IL-12 cytokine family and is comprised of the same EBI3 protein subunit in IL-35. Like IL-12p40, EBI3 expression is much more abundant in macrophages compared with IL-27p28. IL-27 production is clearly dependent on IL-27p28 expression. We did not detect IL-27 production in M2 macrophage culture supernatant following HKC stimulation (Figure S3). However, LPS was able to induce significant levels of IL-27 production, although LPS induced lower EBI3 expression in M2 macrophages when compared with *Candida*. Furthermore, we did not observe an altered LPS induced IL-27 production with increasing doses of *Candida* stimulation (Figure S4). This suggests that *Candida* did not result in suppression of all cytokines, but rather it was specific for IL-12p70 through competition of p35 for dimerisation. Furthermore, the bone marrow derived dendritic cells (DCs) from EBI3 knockout mice produced higher IL-12p70 in response to LPS stimulation, showing that EBI3 plays an intrinsic role in suppressing IL-12p70 production in mice (Figure 7 in USA patent file: Pat No. US2009/022498-A1). We have also shown in this study that knocking down EBI3 by esiRNA rescued the HKC suppression for LPS-induced IL-12p70 production in mouse macrophages (Figure S3).

These results demonstrate for the first time that *C. albicans* induces EBI3 expression in M2 macrophages and blocks LPS-induced M2 to M1 macrophage phenotype transformation. This may serve as one of the mechanisms utilised by *C. albicans* to induce immune tolerance.

## Supporting Information

Figure S1 M2 macrophages expressed significant higher TLR4 levels than M1 macrophages, ** P<0.01.
(PPTX)

Figure S2 **IL-35 and IL-27 did not suppress LPS induced IL-12p70 production by M2 macrophages.**
(PPTX)

Figure S3. **A. EBI3 mRNA knockdown in RAW264.7 cells. B. EBI3 mRNA knockdown rescued HKC induced suppression for LPS stimulated IL-12p70 production.**
(PPTX)

Figure S4 **Heat killed** ***Candida albicans*** **(HKC) alone did not induce IL-27 production in M2 macrophage, and increasing the dose of HKC did not suppress LPS induced IL-27 production.**
(PPTX)
