# Supplementary figures and images for: Lipopolysaccharide-Induced M2 to M1 Macrophage Transformation for IL-12p70 Production Is Blocked by Candida albicans Mediated Up-Regulation of EBI3 Expression
Source: PLoS One. 2013 May 27;8(5):e63967. doi: 10.1371/journal.pone.0063967 (PMC3664618; doi:10.1371/journal.pone.0063967)

## Slide 1
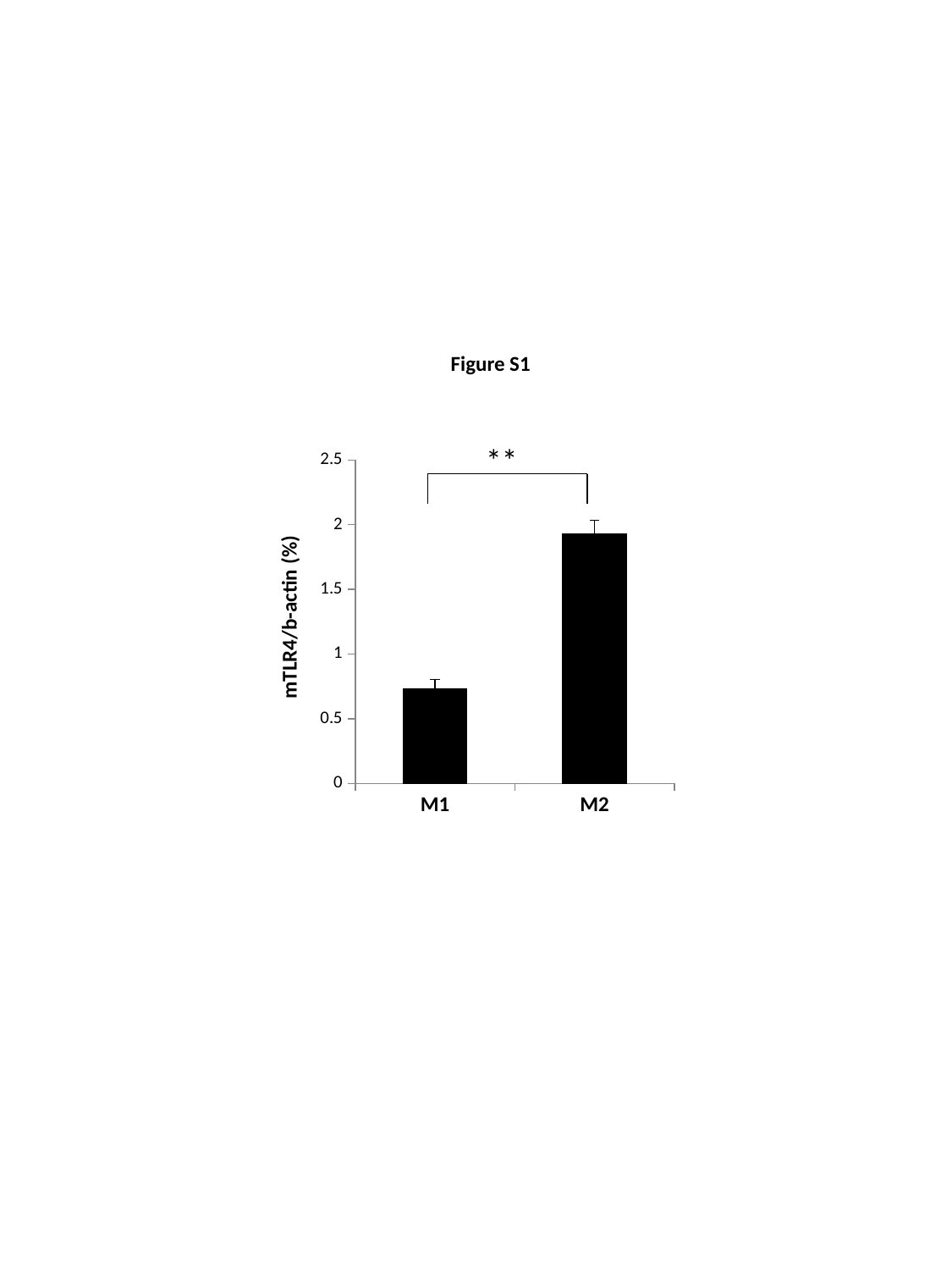

Figure S1
**
### Chart
| Category | |
|---|---|
| M1 | 0.7308073920570352 |
| M2 | 1.9303156823076755 |mTLR4/b-actin (%)

Supplement: Figure S1 — M2 macrophages expressed significant higher TLR4 levels than M1 macrophages, ** P<0.01. (PPTX) [file pone.0063967.s001.pptx]

## Slide 1
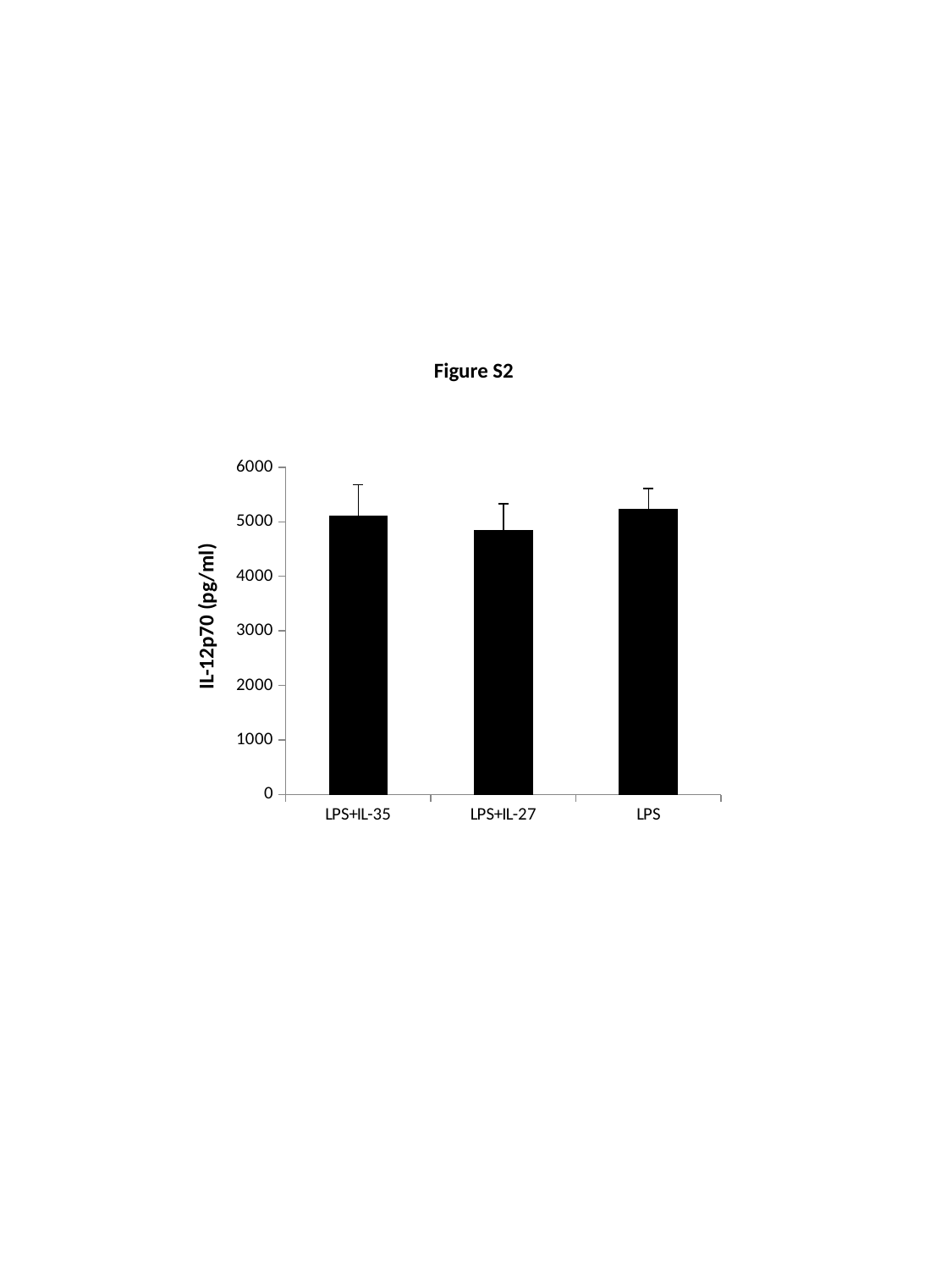

Figure S2
### Chart
| Category | |
|---|---|
| LPS+IL-35 | 5112.564 |
| LPS+IL-27 | 4839.767908923389 |
| LPS | 5226.334860095251 |IL-12p70 (pg/ml)

Supplement: Figure S2 — IL-35 and IL-27 did not suppress LPS induced IL-12p70 production by M2 macrophages. (PPTX) [file pone.0063967.s002.pptx]

## Slide 1
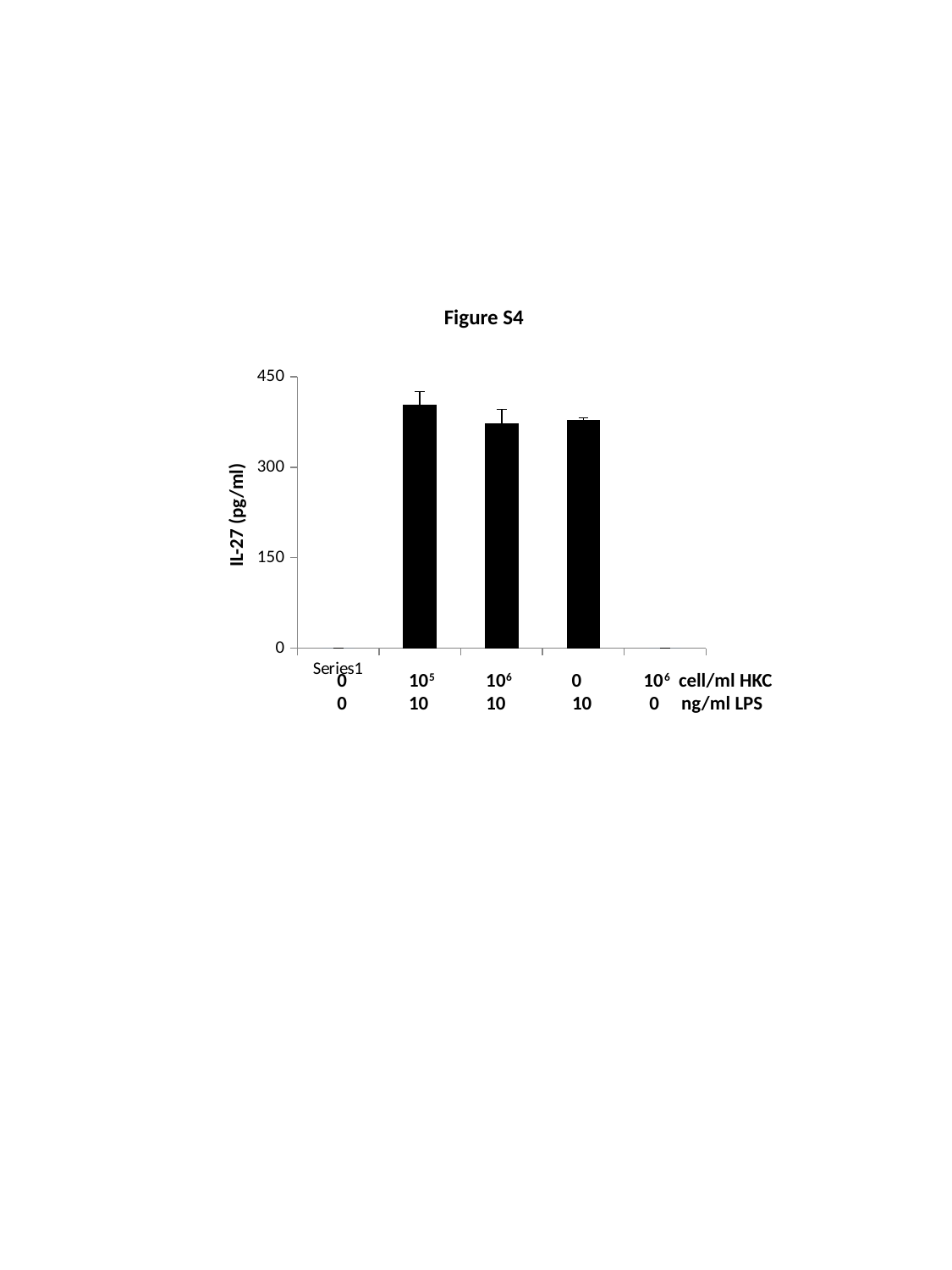

Figure S4
### Chart
| Category | |
|---|---|
| | 0.0 |
| | 403.41499116696673 |
| | 371.93735116498624 |
| | 377.4081096555317 |
| | 0.0 |IL-27 (pg/ml)
0 105 106 0 106 cell/ml HKC
0 10 10 10 0 ng/ml LPS

Supplement: Figure S4 — Heat killed Candida albicans (HKC) alone did not induce IL-27 production in M2 macrophage, and increasing the dose of HKC did not suppress LPS induced IL-27 production. (PPTX) [file pone.0063967.s004.pptx]
