# Supplementary material for: Lipopolysaccharide-Induced M2 to M1 Macrophage Transformation for IL-12p70 Production Is Blocked by Candida albicans Mediated Up-Regulation of EBI3 Expression
Source: PLoS One. 2013 May 27;8(5):e63967. doi: 10.1371/journal.pone.0063967 (PMC3664618; doi:10.1371/journal.pone.0063967)
Supplement: Figure S3. — A. EBI3 mRNA knockdown in RAW264.7 cells. B. EBI3 mRNA knockdown rescued HKC induced suppression for LPS stimulated IL-12p70 production. (PPTX) [file pone.0063967.s003.pptx]

## Slide 1
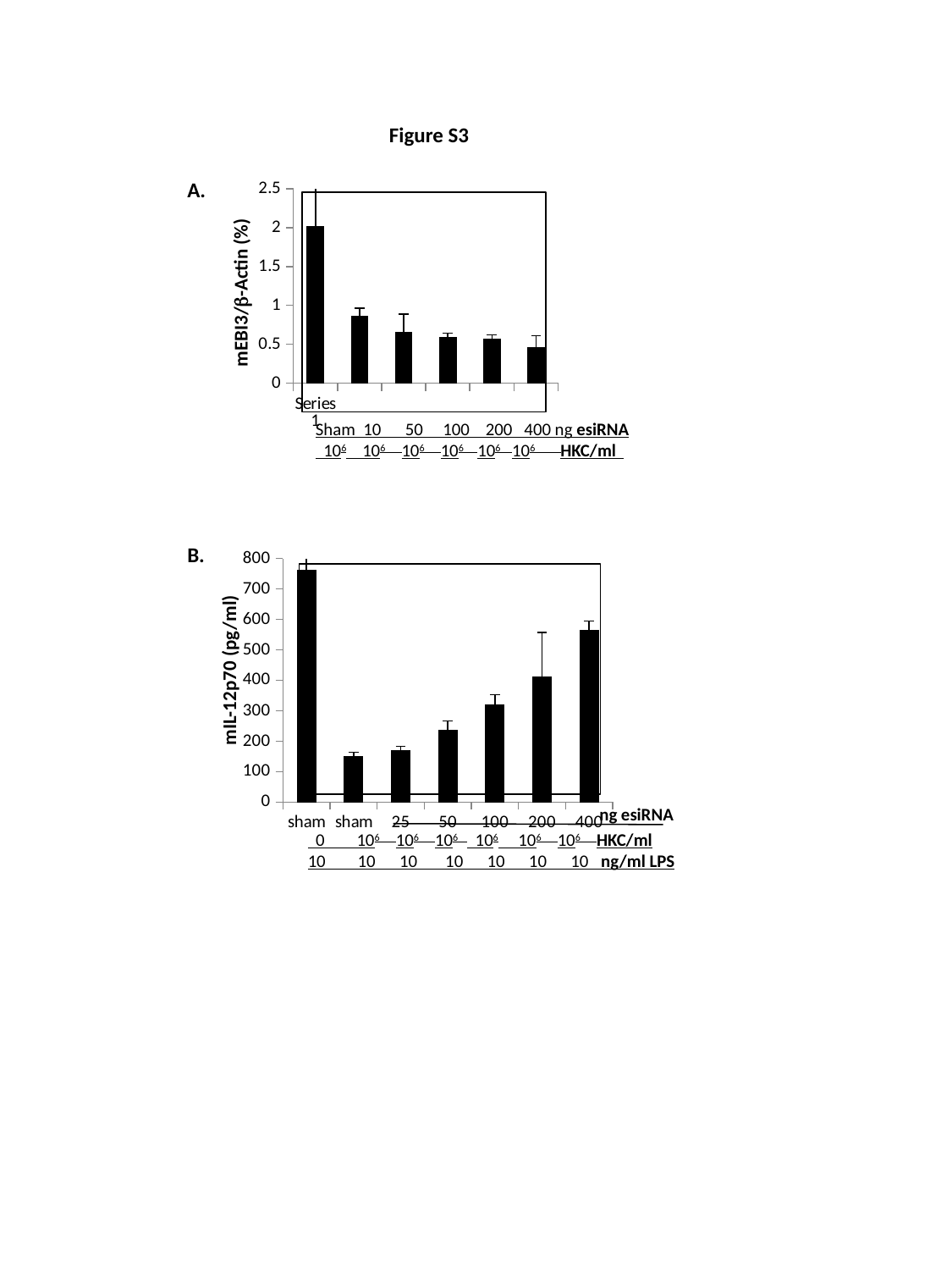

Figure S3
A.
### Chart
| Category | |
|---|---|
| | 2.019301297871085 |
| | 0.8668511500530018 |
| | 0.661519775283225 |
| | 0.5975740990328106 |
| | 0.5705886111758097 |
| | 0.45813865204370174 |mEBI3/b-Actin (%)
Sham 10 50 100 200 400 ng esiRNA
 106 106 106 106 106 106 HKC/ml
B.
### Chart
| Category | |
|---|---|
| sham | 761.2063295660741 |
| sham | 148.97089587941818 |
| 25 | 170.0545726439351 |
| 50 | 237.26891314913658 |
| 100 | 320.40364945221296 |
| 200 | 410.0754401881717 |
| 400 | 562.7399185278381 |
mIL-12p70 (pg/ml)
ng esiRNA
 0 106 106 106 106 106 106 HKC/ml
10 10 10 10 10 10 10 ng/ml LPS
